# Notes and News

**Published:** 1887-10-08

**Authors:** 


### NOTES AND NEWS.

COLLECTED AND COMMUNICATED.

WE have pleasure in being able to announce that we hope to publish next week an article by the Bishop of London, entitled "Healing the Sick."

THE governors of Guy's Hospital have received an anonymous donation of £400 from E. M. G.

MR. J. C. WILLIAMS, of Werrington Park, Launceston, has made the munificent contribution of £1,000 towards the extinction of a debt of £3,000 on the South Devon Hospital.

CAPTAIN PHILIP HAMON has been appointed Director of the Jersey General Hospital, and Mrs. Hamon will occupy the position of Directress.

AT the quarterly meeting of the trustees and governors of the York County Hospital, held on Wednesday, it was resolved that steps be taken for the appointment of a permanent house-steward.

MR. G. A. GEORGE has been elected honorary surgeon to the Dorset County Hospital. The only other candidate was Mr. A Emson, Junior. Mr. George had a majority of 108 votes.

THE *Birmingham Gazette* thus describes the condition of affairs at the Queen's Hospital in that town :—"Unable to keep up with the wants of the times : the work ever increasing : no improvements on any large scale attempted : doctors and nurses cramped for room : sanitary arrangements defective : some of the principal physicians intimating that they will be obliged to discontinue their services unless a remedy is effected and the impediments in the way of the proper performance of their duties removed : year by year the debt increasing." The Mayor, Sir Thomas Martineau, in an energetic and business-like speech, insisted that the people should stir up in themselves that kind of enthusiasm which enabled a preceding generation to place Birmingham in the forefront of practical philanthropy. Will the people do so, as Sir Thomas says they ought to do ?

WE remind our readers that Sir Andrew Clark will preside at the meeting of The Hospitals Association, to be held at the Library of the Society of Arts, John Street, Adelphi, at 8 p.m. on Wednesday next, the 12th inst., when the scheme for a National Pension Fund for Hospital Officials and Nurses will be explained by Mr. Burdett. Everybody interested is invited to attend the meeting and take part in the discussion, as by no other means is it possible to formulate a workable scheme. Any lady or gentleman interested in the subject should at once apply to Mr. Howard J. Collins (Secretary), The Hospitals Association, Norfolk House, Norfolk Street, W.C., for cards of invitation.

THE contract recently entered into by the authorities of the Isle of Man Fever Hospital for the erection of the new building was, by a resolution passed at their last meeting, repudiated.

COPIES of the annual report of The Hospitals Association have been sent to all members. Any member not having received a copy will oblige by communicating with the Secretary. A copy of the annual report has also been sent to the secretary of each of the principal hosptals and infirmaries throughout the kingdom. It is requested that each secretary will be so good as to forward in return a copy of the last published report, together with the laws and regulations of his institution, to the Secretary, The Hospitals Association, Norfolk House, Norfolk Street, W.C. Mr. Collins will be glad to send copies of the report of the Association on application to any philanthropic nursing or medical institution.

NEARLY £15,000 has already been subscribed towards the building fund of the proposed new hospital for Glasgow. Comparing the rapid progress of this fund with the creeping pace of that for the new buildings of the Great Northern Central Hospital in London, the conclusion arrived at is that the Glasgow people are much more generous than those of North London, or that the managers of the Glasgow fund are men of greater energy and capacity than the North London managers.

THOSE who are familiar with the amusing play, "Our Boys," will know, on the unimpeachable authority of Mr. Middlewick, that Dorset is a county famous for butter. The Dorset County Hospital has just invited tenders for the supply of that article for a period of several months. A contract has been entered into by which the hospital will be supplied at 1s. 2d. per pound. Not a single farmer sent in a tender, although it is said many of them send a weekly supply of butter to London, for which the price they receive is 10d. It seems that farmers are just as stupid and helpless in Dorset as in other counties.

IT often takes a long time for new institutions to reach out-of-the-way places. Salisbury is not so very remote from the centre of modern life, yet last Saturday was the first "Hospital Saturday" the ancient city has witnessed. Though late in commencing, the friends of the hospital seem to have taken up the work with vigour and determination. A committee of ladies has been appointed, and some of these visited various places of business in the city, with the result that the handsome sum of forty pounds was speedily realised. Hospital Sunday was observed the day after the Saturday collection. Many experienced people think it is a mistake to have the two collections so near each other. A few weeks, or even two or three months' interval, are said to be favourable to both.

The following vacancies are announced this week, the amount of the salary being placed in each case after the name of the institution :—

**Resident Medical Officers.**

Brompton Hospital for Consumption. Resident Clinical Assistant.
North-West London Hospital. Assistant Resident Medical Officer.
Victoria Park Chest Hospital. Resident Clinical Assistant.

**Non-resident Medical Officers.**

Bristol Royal Infirmary. Obstetric Physician.
Royal Berks Hospital, Reading. Honorary Physician.

**Matrons.**

Bradford Infirmary, Yorkshire.—£50.
York Asylum, Bortham. Matron's Assistant.

**Trained Nurses.**

Barnsley Fever Hospital.—£45 10*s*.

Cottage Hospital, Ballymena, Ireland.

Mildmay Mission Hospital, Turville Street, Shoreditch. Night Nurse. £25.

Nursing Institution, Ryde, I. W. Several. Apply Superintendent.

Royal Portsmouth Hospital. Head Nurse.—£25, uniform.

St. Luke's Institution for Nurses, Oppidans Road, N.W. Several.

**Probationers.**

Chelsea Infirmary, Cale Street. One.
Newark Hospital, Notts. One.
Poplar Hospital for Accidents. One.

---

The house-to-house collection at Lincoln this year amounted to £114 4*s*. 8*d*., as against £124 17*s*. 9*d*. last year, and £134 9*s*. 2*d*. in 1885. At this rate the house-to-house collection will be dead and buried in twelve years. "Alas for the rarity of Christian charity under the sun."

---

The Corporation of Sheffield has been served with a "writ" to restrain them from building a small-pox hospital in the parish of Totley. It may not be either necessary or right that Sheffield should build an infectious diseases hospital at Totley; but it is both necessary and right that they should build one somewhere. The question is—where?

---

Are there no poor at Folkestone needing hospital aid? There cannot be, or the congregation at the parish church would never have allowed it to be publicly proclaimed in the newspapers that the offertory on Hospital Sunday, after a sermon by the vicar, amounted to no more than £7 18*s*. 4*d*.

---

The development of institutions under the pressure of their environment is well illustrated by the recent action of the honorary secretaries of the Cardiff Infirmary, Mr. G. T. Coleman and Mr. F. W. Lock. They have despatched upwards of 4,000 circulars, accompanied by collecting-books, to employers of labour and tradesmen in the district, bespeaking their aid in making the annual collections from the working-classes. The area of the appeal has this year been enlarged so as to embrace a number of the smaller tradespeople, who it is believed are very willing to give small sums, but have not previously had such an opportunity as the Saturday fund affords them. If Mr. Herbert Spencer desires to see his own principles in actual operation on a considerable scale, he cannot do better than study the hospital system.

---

No one can say that the Charity Organisation Society is greatly beloved by the struggling poor. It takes rank in their estimation with the rate-collector, the gas-man, and the Christmas dun. Its functions are mainly inquisitional, and each of its agents is looked upon as a kind of ubiquitous Paul Pry. Nevertheless it does much good work, and may yet do more if the public can be prevailed upon to augment its resources. At Derby the local branch is instituting a fund for sending hospital and other convalescents to selected homes, where they recover their full strength before being called upon again to march in the battle of life. The Duke of Devonshire has issued an appeal on behalf of the movement, and Mr. W. H. Worthington starts the subscription list with a gift of £20.

A handsomely-bound volume containing sixty-five Turner engravings was presented to Dr. Thomson by the Admission Committee on his resignation of the office of Honorary Surgeon to the Oldham Infirmary.

---

Harvest thanksgivings are now numerous. It may be hoped the hospitals will not be forgotten when the fruit is distributed. We do not hear of many consignments of game finding their way to the wards. Are sportsmen lazy or forgetful?

---

Mr. J. W. Maclure, M.P., on behalf of himself and several friends, has offered to build and equip a cottage hospital containing twenty beds at Hulme, near Manchester, on condition that the managers of the Manchester Royal Infirmary will be responsible for its direction and maintenance when completed.

---

The town of Wakefield, containing 40,000 inhabitants, is proposing to spend a thousand pounds on a fever hospital. What kind of building and sanitary arrangements do the sages of Wakefield expect to get for a thousand pounds? The spinning trade is surely very depressed, or the Town Council very stupid. Yorkshiremen were not wont to be so poor-spirited.

---

A special floral and musical service was held on Sunday at the Agricultural Hall, Islington, on behalf of the Morley Convalescent Home at St. Margaret's. The choir of the Finsbury Park Young Men's Christian Association and the Wellington Light Cavalry Band were present. The Rev. W. Evans Hurndall, M.A., of Bow, and the Revs. Dr. Thain Davidson and Matthew Smith also took part in the proceedings. The audience was large.

---

The dissenting places of worship, with one exception, appear to have taken no part in the Hospital Sunday Collection at Bournemouth this year. Such, at least, is a statement for which the *Bournemouth Guardian* is responsible. We only hesitate to express an opinion on this statement because we can hardly believe it to be true. Can there be any possible explanation which the *Guardian* has not seen fit to publish?

---

We have received a communication from Miss Pringle with reference to an article in our issue of the 17th September, in which she says that while appreciating the kindness of our intentions, she feels distressed that she should have been made the subject of notice. She requests us, in justice to St. Thomas's, her Alma Mater, to say that some points of importance in our paragraph are inaccurate, and that she would desire to describe the change which she has made as a move from one Home to another, both rich in privileges. We gladly publish this disclaimer, but we desire it to be understood that there was no intention whatever to place the claims of one hospital in contrast with those of another. Anyone who is familiar with the two institutions in question knows that in each the post of matron is one of the highest dignity and importance. We thought it honourable to Miss Pringle to be willing to give up friends, associations, and known duties for a new and untried position, where many at least of her former happy circumstances could not but be wanting. We do not doubt that she will find friends and other compensations at St. Thomas's; but we cannot think otherwise than that she has followed the call of duty, and not of mere personal advantage, in electing to come from Edinburgh to London.

Three little maidens, whose united ages are twenty-five years, have held a "bazaar," all by themselves, in aid of the Bradford Infirmary. They bought the raw material, made the goods and sold them, without the aid of a committee. Ten-and-sixpence was the grand total of their sales, and this they handed over, deducting nothing for expenses, to the Hospital treasurer. These three little women, who all deserve to get good husbands when they grow up, live in Bowling, the poorest and blackest part of all Bradford. Who will paint their portraits?

The Town Council of Colchester claim £358 from the Hospital as its proportion for the making of the public road which passes the building. The Institution is in straits for want of means, and this Shylock of a Town Council insists upon its pound of flesh. Can the Rev. W. J. Irvine not persuade the local statesmen to take a public and patriotic view of the matter? They might very well celebrate the Royal Jubilee by sending in to the hospital authorities a discharged account.

An average weekly income of £48 18*s.* 1*d.* has been paid over to the treasurer of the Leeds Infirmary by the Workpeople's Medical Charities Committee since the beginning of the year, or a grand total to date of £1,869 17*s.* 11*d.* So far as we know, the workpeople of no town of the same magnitude have done anything like this on behalf of the local hospital. This is the result of goodwill conjoined with intelligent and systematic effort.

Should not Hospital Saturday be appointed for summer instead of autumn? The weather is now very pleasant for those who are in rapid movement, but excessively cold for any who are compelled to stand still. At Eastbourne the annual collection has been greatly diminished, and it is said the lateness of the date is the cause of the falling off. Besides this untoward result, the young ladies who took charge of the street boxes had good reason to complain of the severe ordeal which a sense of duty had imposed upon them. In our opinion Hospital Saturday will never be a success until it is confined to the workshops and establishments, and so made into a *bonâ fide* working-class movement.

The following account of a harvest festival, by the Rev. G. W. Rolfe, rector of Swanton Novers, appeared in the *Pall Mall Gazette*:—

"My village has set a good example 't' year,' as we say in Norfolk. A fortnight ago the happy thought came with the morning pipe that a joint-stock church and chapel festival was alike desirable and feasible. Away I went to one of the worthy deacons-in-chief and boldly popped the question, 'Will you join hands?' He was at once agreeable, and accepted, subject to the approval of his colleagues. This was readily given. The joining of choirs was the first step, and all the singers, dissenters and assenters, came to the two practices preceding the service. The harvest hymns from the first were a joy to them, though the canticles and special psalm were rather a difficulty from the musical point of view. But 'We shall get "t'reugh" it all right, I think, sir,' was at last their triumphant conclusion from their own performances. To the church on Saturday flocked the villagers, laden with fruits of the earth, till the chancel was a perfect picture, and every available nook and cranny bore evidence of the bountifulness of the harvest. On Sunday streams of people poured in, filling all the seats and every overflow bench and chair. 'All sorts and conditions of men' they were, from peer to peasant, from hoary veteran to helpless babe. Every family in the village and every sect were represented: all our farmers and nearly all our labourers were there. The service went with tremendous swing, everybody 'singing' his best, and the Old Hundredth at the end of the service was never given more heartily in a Yorkshire 'shout' of praise. The collection (for hospital and nurses' home) realised 151 copper and sixty silver coins,—not bad for a little village of 287 inhabitants; and throughout the day the grand total was £4 11s. 0½d. On Monday we sent off five big boxes of many and varied offerings to the hospital, amounting to nearly a quarter of a ton, and so ended a *real* parish thanksgiving."

Mr. Ellis Lever, of Manchester, writes under a misapprehension that we object to the bringing of sea-water to London for sanitary purposes. We published a note a short time ago from a contemporary, which did not quite coincide with Mr. Ellis Lever's view of the matter, not, however, with the intention of opposing Mr. Lever, but of calling further attention to the question. It is a subject on which there may be a variety of opinions, and it is only by discussion that sound working principles can be arrived at. If Mr. Lever has any convincing arguments to use, we shall be glad to give him space to enforce them.

We have received an inquiry from one of the Australian Colonies respecting convalescent homes to accommodate over sixty inmates, but to be constructed on the separate-cottage system, with a few, say from four to eight residents in each building. We would refer our correspondents to Mr. Ernest Morgan, the secretary of the Royal National Hospital for Consumption at Ventnor, the London offices of which are at 34, Craven Street, Charing Cross, W.C. That institution is the one which most nearly resembles the kind of home about which information is desired. A visit to Ventnor would no doubt be the shortest and best way to profit by the experience which the practical working of this valuable institution can supply. The separate-cottage system has not met with much encouragement, owing no doubt to the difficulties of administration, and the increased original and after cost.

Miss Fannie Surtees is not pleased that a suggestion of hers for the benefit of the Exeter Hospital has fallen upon stony ground. She desired that as the recent dreadful calamity in that city had put an unusual strain upon the resources of the hospital, a special effort should now be made to recruit their resources, and she offered to head a subscription list. The suggestion was good and the offer generous; but perhaps the former was a little ill-timed. Just at present the sympathy all goes with the sufferers, and the practical help will undoubtedly follow the sympathy. In a few months, when the attention of the public is a little less concentrated upon the sufferers, a judicious appeal on behalf of the hospital might be printed, with the convincing argument that if there had been no such institution in Exeter at the time of the fire, the city would have been absolutely helpless in presence of one of the most terrible tragedies of modern times.

### THE MEDICAL SCHOOLS.

St. Mary's Hospital.—The winter session was opened on Monday with an address by Mr. Anderson Critchett, an abstract of which we publish elsewhere.

St. George's Hospital.—Mr. C. J. Dent, F.R.C.S., gave the opening lecture at St. George's, treating, among other things, of the nature and significance of pain.

University College Hospital.—Dr. Radcliffe Crocker, F.R.C.P., was the speaker at the opening of the winter session at University College Hospital. He spoke strongly against the advertising arts employed by some of high professional qualifications, who are intent upon pushing themselves prematurely into consulting practice. A kind of professional boycotting was a remedy which he thought might be tried.

Westminster Hospital.—The session was opened by Dr. Sturge, with an address on the present condition and future prospects of medical education.

King's College Hospital.—The Earl of Selborne presided and distributed the prizes won by the successful students.

Middlesex Hospital.—The Lord Mayor, distributing the prizes at this hospital, counselled the students to make full use of the advantages offered in the course of study they were now commencing.

